# Women’s awareness regarding the use of dental imaging during pregnancy

**DOI:** 10.1186/s12903-021-01726-6

**Published:** 2021-07-20

**Authors:** Lina Bahanan, Abdulrahman Tehsin, Reyouf Mousa, Mohammed Albadi, Mohammed Barayan, Emad Khan, Hanadi Khalifah

**Affiliations:** 1 Department of Dental Public Health, Faculty of Dentistry, King Abdulaziz University, Jeddah, Saudi Arabia; 2 Dr. Samir Abbas Hospital, Jeddah, Saudi Arabia; 3 My Clinic KSA Medical Centre, Jeddah, Saudi Arabia; 4 Medical services of Saudi Royal Guard, Jeddah, Saudi Arabia; 5 Oral and Maxillofacial Radiology Division, Faculty of Dentistry, King Abdulaziz University, Jeddah, Saudi Arabia

**Keywords:** Dental imaging, Pregnancy, Awareness, Survey, Oral health

## Abstract

**Background:**

There is often anxiety among pregnant women about dental imaging during pregnancy. This may hinder some women from seeking dental treatment during pregnancy and consequently, may negatively affect the oral health of the mother and fetus. This study was conducted to assess women’s awareness regarding the use of dental imaging during pregnancy.

**Methods:**

In this cross-sectional study, the electronic distribution of structured questionnaires was done via social media. The self-administered questionnaires contained questions related to women’s knowledge regarding the radiation protection measures during dental imaging, the safest period for dental imaging, the type of radiographs that can be acquired during pregnancy, and the possibility of radiation-induced malignancy and fetal malformation as a result of dental imaging.

**Results:**

In total, 410 completed questionnaires were received and analyzed. More than half of the participants were 30–49 years of age. The majority of the participants (91%) demonstrated poor knowledge concerning dental imaging. Only 4% reported that pregnant women can have dental imaging during any trimester. The majority believed that panoramic images and cone-beam computed tomography should not be acquired during pregnancy. The majority also believed there is a high risk of congenital malformation due to dental imaging and were unsure about the oncogenic risks.

**Conclusions:**

Our study suggests that there is insufficient knowledge about dental imaging safety during pregnancy. This misconception may have a direct impact on the attitude toward seeking dental care. Therefore, community awareness initiatives aimed at informing our society about radiation exposure, safety, and required protection measures are critical.

**Supplementary Information:**

The online version contains supplementary material available at 10.1186/s12903-021-01726-6.

## Background

Dental imaging is an essential tool for the diagnosis of oral diseases, management, and assessment of treatment outcomes. For many years there has been a misconception when it comes to diagnostic dental imaging and pregnancy. The American Dental Association and the American Congress of Obstetricians and Gynecologists state that pregnant women can take dental radiographs, at any trimester during the pregnancy, with the application of radiation protection measures to keep the dose as low as reasonably achievable [1, 2]. The lack of awareness regarding the safety of dental imaging hinders pregnant women from seeking dental treatment. It is crucial that pregnant women maintain their dental and oral health since the mother’s oral health is linked to the offspring’s oral health [3–5].

Several studies have demonstrated that dental students and dentists have a shortage of knowledge concerning diagnostic imaging and radiation safety [6–9]. Physicians’ perceptions about the teratogenic effects and risks from various imaging examinations have been investigated. Studies have shown that misperceptions are high, which may have an adverse effect on patient care [10, 11]. Considering the lack of available literature, this study was conducted to assess women’s awareness regarding the use of diagnostic dental imaging during pregnancy. Knowledge about radiation safety and protection during pregnancy is a vital element to enable women to safely seek dental treatment during pregnancy. We hypothesize that women have poor knowledge regarding diagnostic dental imaging during pregnancy.

## Methods

### Study design and participants

This research was reviewed and approved by the Institutional Review Board at King Abdulaziz University Faculty of Dentistry, Jeddah, Saudi Arabia (#238-04-21). The study followed the Strengthening the Reporting of Observational Studies in Epidemiology (STROBE) checklist.

This cross-sectional study was carried out from March 2020 to August 2020. We conducted a self-administered questionnaire to evaluate the knowledge and perceptions of women regarding the precautionary measures of diagnostic dental imaging during pregnancy. A non-probability snowball sampling technique was used to recruit the study population through social media platforms such as WhatsApp and Twitter. We included female participants living in Saudi Arabia, aged 18 years or older.

### Sample size

We calculated the sample size using the online *Raosoft sample size* calculator [12]. The calculation was based on a population size of 14.2 million women, 50% response distribution, with a confidence level of 95%, and a margin of error of 5%. The minimum required sample size for this study was 385 participants. The final number of recruited participants was 410 women to account for any missing data or non-response rate.

### Questionnaire design

The questionnaire was developed by two oral and maxillofacial radiologists based on common misconceptions among patients visiting dental clinics about radiographic examinations and pregnancy. The questionnaire was in the Arabic language, pre-structured and anonymous. It was assessed by two experts in the field of oral and maxillofacial radiology to ensure content validity then it was pre-tested through a selected sample of dentists to ensure clarity and face validity. Prior to launching the study, it was piloted with 15 women from the target population. The questionnaire was developed using Google forms and distributed through social media platforms such as WhatsApp and Twitter.

The cover page of the questionnaire included an explanation of the study’s objective, assure confidentiality and voluntariness, and provide contact information of the principal investigator. The questionnaire was composed of two sections. The first section was about sociodemographic characteristics. The second section was composed of nine multiple-choice questions about the awareness regarding the safe use of dental imaging for pregnant women. Respondents were asked “when should a woman inform the treating dentist about pregnancy”, “which trimester of pregnancy is safe for dental imaging”, “what should a pregnant woman wear while acquiring dental radiographs”, and “who should hold the image receptor inside the patient mouth during image acquisition”. Respondents were asked whether the radiation dose to which the body is exposed from a single intraoral dental radiograph is less, more, or equal than the daily background radiation. Also, participants were asked if it is allowed to take a panoramic radiograph or Cone Beam Computed Tomography (CBCT) for dental purposes during pregnancy. Lastly, they were asked about the possibility that the fetus will develop birth defects or cancer due to the use of dental imaging during pregnancy. All questions and options can be found in Additional file 1. The total possible score for the knowledge questions was 9 points and a score of 1 was given for correct answers, while a score of 0 was given for incorrect answers. The level of knowledge was then categorized according to the number of correct answers (poor: 0–3, fair: 4–6, good: 7–9).

### Study variables

Demographic characteristics included age (< 30, 30–39, 40–49, and ≥ 50), marital status (single or married), and education (less than high school, high school graduate, college, higher education). The participants were asked if they work or study in the medical field (yes or no).

### Statistical analysis

All statistical analyses were conducted using SAS 9.4 software (SAS Institute Inc., Cary, NC, USA). Univariate analyses, such as frequencies and percentages, were used to report the characteristics of the participants, as well as the perceptions and knowledge of the participants. Bivariate analyses were used to compare the knowledge of women against the characteristics of the study sample. Multinomial logistic regression analysis was conducted to evaluate the associations between the different predictors and levels of knowledge. Covariate variables were selected from previous literature [13]. We adjusted for age, education, marital status, and whether the participant was studying or working in the medical field. The significance level was set at *P* < 0.05.

## Results

This study included 410 women with no missing data on the study variables. More than 50% of the participants were aged 30–49 years, and most were married. More than two-thirds of the participants had a college degree and most did not study or work in the medical field. Only 8% had fair knowledge, while 91% had poor knowledge regarding dental imaging during pregnancy (Table 1). Among the respondents, 41% were not aware of the radiation protection measures during dental imaging. The majority of respondents reported that pregnant women should inform the radiologist if she is pregnant or expecting. Only 4% reported that pregnant women can have dental imaging during any trimester. The majority believed that two layers of lead aprons are needed. Only 11% reported that the radiation dose from a single dental radiograph is less than the natural background radiation. Regarding the type of radiographs that can be acquired during pregnancy, only 13 and 6% responded that panoramic radiographs and cone-beam computed tomography (CBCT) are not contraindicated, respectively. Only 11% of the participants reported that the risk of fetal malformation and cancer due to radiation exposure is very low (Table 2).

**Table 1:** Characteristics of the study population (n = 410)

Characteristics	Full sample n (%)
*Age*	
< 30	76 (18.5)
30–39	118 (28.8)
40–49	111 (27.1)
≥ 50	105 (25.6)
*Marital status*	
Single	81 (19.8)
Married	329 (80.2)
*Education*	
Less than high school	11 (2.7)
High school graduate	66 (16.1)
Collage	284 (69.3)
Higher education	49 (11.2)
*Work or study in the Medical field*	
Yes	67 (16.3)
No	343 (83.7)
*Level of knowledge*	
Good (score 7–9)	2 (0.5)
Fair (score 4–6)	33 (8.1)
Poor (score 0–3)	375 (91.5)

**Table 2:** Knowledge about the precautionary measures of taking dental radiographs during pregnancy

Knowledge items	Frequency (%)
Pregnant women should inform the radiologist if she is pregnant or expecting	355 (86.6)
Pregnant women can take radiographs at any trimester	16 (3.9)
Pregnant women should wear a lead apron and thyroid collar while taking a dental radiograph	102 (24.9)
The radiation dose during pregnancy is less than the usual dose	47 (11.5)
Intraoral films should be held by the film holder	115 (28.1)
Pregnant women can take a panoramic radiograph	54 (13.1)
Pregnant women can take CBCT	24 (5.9)
The risk of cancer among infants due to radiation exposure is very low	46 (11.2)
The risk of fetal malformation due to radiation exposure is very low	47 (11.5)

Multinomial logistic regression analysis was conducted to investigate the association of the predictors and their level of knowledge. The knowledge questions assessed the knowledge and awareness of pregnant women regarding the precautions and misconceptions of taking diagnostic dental imaging during pregnancy. Working or studying in the medical field was the only significant predictor that was associated with the level of knowledge (odds ratio [OR]: 7.9, *P* < 0.05). Working or studying in the medical field increased the odds of having better knowledge about the precautions of taking dental radiographs during pregnancy. Age, marital status, and education levels were not associated with the level of knowledge (Table 3).

**Table 3:** Multinomial logistic regression showing predictors of knowledge level

Characteristics	Odds ratio (OR)	95% confidence interval
*Age*		
< 30 30–39 40–49 ≥ 50	Ref 0.6 2.2 0.7	Ref 0.2–1.9 0.5–9.5 0.2–0.8
*Marital status*		
Single Married	Ref 1.7	Ref 0.6–4.6
*Education*		
Less than high school High school graduate Collage Higher education	0.1 0.7 0.4 Ref	0.1–0.8 0.1–4.1 0.1–1.6 Ref
*Work or study in the medical field*		
Yes No	Ref 7.9	Ref 3.2–19.2*

*P < 0.05

## Discussion

Dental imaging is a key part of dentistry as it is a key diagnostic tool and images can easily be shared with colleagues for confirmation of diagnosis and advice. Traditionally, dental imaging is avoided during pregnancy, specifically during the first trimester, to protect the developing fetus. However, oral health may be affected throughout the pregnancy and radiographic examinations for a proper diagnosis and management of various dental conditions might be needed. Out of fear and anxiety concerning the risk of cancer or genetic malformations, pregnant women are generally reluctant to have dental imaging, which may in turn delay necessary treatment and could negatively impact the health of both the mother and fetus. This study was conducted to investigate women’s awareness regarding the use of diagnostic dental imaging during pregnancy.

Dental imaging is safe during pregnancy as long as the radiation protection measures have been applied [14]. Radiation doses can be substantively reduced by various measures, such as radiographic selection criteria, a lead apron with a thyroid collar, high-speed film or digital imaging, and most importantly, rectangular collimation [15]. For bitewing and full mouth radiographs, the use of digital sensors or an F-speed film in combination with rectangular collimation was found to reduce radiation exposure by a factor of 10 [16].

Our study demonstrated that participants have poor knowledge regarding radiation safety during pregnancy. A very small percentage of the participants were aware that dental imaging can be acquired during any trimester with the application of radiation protection measures. Around two-thirds of the participants were not sure or thought it was contraindicated in all trimesters. The majority believed that panoramic radiographs and CBCT are contraindicated during pregnancy. Less than half of the participants were not sure about the radiation protection measures that have to be applied during dental imaging. A large number of participants had misconceptions, like the existence of a specific lead apron for pregnant patients or that two layers of lead aprons are needed. The position statement of the American Association of Physicists in Medicine (AAPM) cited the justifications for disregarding the fetal and gonadal lead shield claiming that the risks from diagnostic imaging are “minimal to non-existent” [17]. While it was suggested that lead shielding is not necessary, it provides a sense of safety and comfort to the patient.[17–19].

A possible explanation is the lack of public radiation awareness programs. Also, it is possible that patients are not informed about the radiation safety and risks by their treating dental practitioners. A study by Al Faleh et al., reported that almost 40% of the patients were not informed about the radiation hazards by their dentists [13]. More than half of the patients never inquired about safety measures before undergoing imaging. Furthermore, the patients’ lack of knowledge could be a reflection of insufficient knowledge among treating dental practitioners. An extensive literature review revealed that there is a worldwide concern about dentists’ knowledge regarding dental imaging during pregnancy. Several studies have revealed that the knowledge of dental students, interns, and dentists on ionizing radiation and radiation protection is very poor [6, 8, 9, 20–22]. In a study by Aboalshamat et al., 67% of dentists considered periapical radiographs safe only during the second trimester. Panoramic radiographs were considered contraindicated during pregnancy by 69% of the participants [21]. Bedre and Sharma found that only 2% of dentists knew that dental imaging is safe in all trimesters and 44% thought it was unsafe in all trimesters [20]. Another study found that half of the Jordanian dentists considered that panoramic radiographs are contraindicated during pregnancy and less than one-third did not know if they are safe [8]. Llea et al. found that more than two-thirds of dentists would acquire dental radiographs only for emergency purposes [6]. It is also possible that dental professionals are unaware about the significant dose reduction associated with digital imaging in comparison to the conventional film. Lack of knowledge could lead to increase anxiety of both dentists and pregnant women seeking dental treatment during pregnancy. Therefore, continuous professional education is crucial to raise dentists’ awareness about the radiation dose from various dental imaging techniques and dose reduction measures.

Knowledge about radiation doses from dental imaging relative to the background radiation dose was also poor. The majority of participants were not sure how the dose from a periapical radiograph compares to the background radiation dose. In comparison to the natural background radiation exposure, the dose from a single bitewing radiograph acquired with a photostimulable plate and a rectangular collimator is less than one day of background radiation [16]. According to the National Council on Radiation Protection and Measurements Report No. 177, the fetal dose from full-mouth intraoral radiographs is 4–6 times less than the exposure to normal background radiation over the nine months of pregnancy [23].

Regarding congenital malformations, a very small percentage responded that such risks are not associated with dental imaging, whereas more than half of the participants believed that the risk of radiation-induced congenital malformations from dental imaging is high. Similarly, Razi et al. found that only 28% of the dentists were aware that radiation doses from diagnostic imaging do not result in congenital malformations or fetal mental retardation [9]. Concerning fetal malformation, the International Commission for Radiation Protection states that the fetal absorbed dose would have to exceed the threshold dose of 100–200 mGy or higher. This is far more than the fetal absorbed doses from diagnostic imaging, as well as nuclear imaging. In both human and animal studies, there is no evidence that the range of radiation exposure from diagnostic imaging (i.e., less than 50 mGy) is linked to an increased risk of teratogenic effects [14, 24, 25].

The risk of childhood cancer is difficult to estimate from low-level exposures, such as dental imaging [16]. Our participants were unsure about the oncogenic risks from dental imaging. Almost one-third of the participants believed that the risk is high, one-third believed that the risk is low, and the remaining participants believed the risk does not exist. The perception that radiation can cause cancer is derived from studies of the survivors of the Hiroshima and Nagasaki atomic bombs and other cohort studies. However, epidemiologic studies have failed to find an association between the radiation dose and the cancerogenic effects [16, 24, 25]. It has been estimated that the fetal dose from a single CT examination of the head ranges from 0 to < 0.005 mGy [24, 25]. Although this knowledge may reassure pregnant women and dental professionals about the safety of dental imaging, it is still prudent to use cautious clinical management and ensure that the dose is kept as low as reasonably achievable.

This study has several limitations. This was a cross-sectional study that does not imply causality. In addition, we used snowball sampling, which undermines the ability to generalize the results to the population. Moreover, this study may be prone to self-selection bias due to the nature of recruiting participants which may jeopardize both internal and external validity. Another limitation is that we did not ask whether or not the woman has had children. Women with children might have better knowledge due to their experience from previous dental visits during pregnancy. Future research is warranted to assess obstetricians’ perceptions of radiation dose and risk associated with various dental imaging during pregnancy. Moreover, we propose further research to evaluate the effectiveness of an educational intervention tailored to educate the public about radiation dose and risk versus benefit. Additionally, a prospective longitudinal study to evaluate the possible role of maternal education from both dentists and obstetricians on the oral health status of pregnant women.

## Conclusions

Our study demonstrated that there is insufficient knowledge in our population regarding the safety of dental imaging during pregnancy. Women’s perceptions of the risk from dental imaging are unrealistically high. This misperception could lead to anxiety and a delay of necessary dental treatment. Since the awareness of participants has a direct effect on their behavior and attitude toward dental care, it is crucial to establish community awareness campaigns that aim to educate our society about the radiation doses, safety, and necessary protective measures. Dental professionals must reassure pregnant patients about the safety of dental imaging during pregnancy and explain its benefits and risks.

## Supplementary Information

**Additional file 1**. The file contains the questionnaire that participants filled out in the study.

## Data Availability

The datasets generated during and analyzed during the current study are not publicly available due to confidentiality agreements but are available from the corresponding author on reasonable request.

## Abbreviations

STROBE: Strengthening the Reporting of Observational Studies in Epidemiology
CBCT: Cone-beam computed tomography
OR: Odds ratio
mGy: Milligray
