# Supplementary material for: Women’s awareness regarding the use of dental imaging during pregnancy
Source: BMC Oral Health. 2021 Jul 20;21:357. doi: 10.1186/s12903-021-01726-6 (PMC8290609; doi:10.1186/s12903-021-01726-6)
Supplement: Supplementary file 1 — Additional file 1. The file contains the questionnaire that participants filled out in the study. [file 12903_2021_1726_MOESM1_ESM.docx]

**Women’s Awareness Regarding the Use of Dental Imaging During Pregnancy**

First section: Demographics

Age

- Below 30 years
- 30-39 years
- 40-49 years
- ≥ 50 years

Marital Status

- Single
- Married

Educational Level

- Less than high school
- High school graduate
- Collage
- Higher education

Do you work/ study in the medical field?

- Yes
- No

Second Section: Knowledge

1) When should a woman inform the treating dentist about pregnancy?

- If she is pregnant
- If she might be pregnant
- Both

2) Which trimester of pregnancy is safe for dental imaging?

- Not allowed
- In the first trimester
- In the second trimester
- In the third trimester
- In any trimester
- Not sure

3) What should a pregnant woman wear while acquiring dental radiographs?

- Lead apron with a thyroid collar
- Two layers of lead apron
- Lead apron intended for pregnant women
- Not sure

4) The radiation dose to which the body is exposed after taking a single intraoral dental radiograph is considered……… the daily background radiation

- Less than
- Equal to
- More than
- Not sure

5) Who should hold the image receptor (film) inside the patient mouth during image acquisition?

- Dentist
- Dental assistant
- Patient with his finger
- A specific holder
- Not sure

6) Is it allowed to take a panoramic radiograph (image shown below) during pregnancy?

- Yes
- No
- Not sure


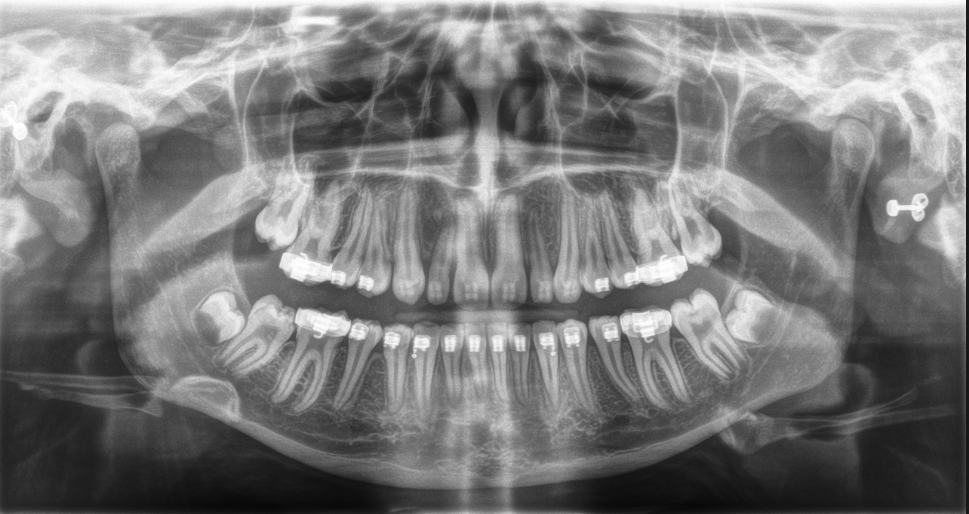


7) Is it allowed to take Cone Beam Computed tomography (3D imaging) for dental purposes during pregnancy?

- Yes
- No
- Not sure

8) The possibility that the fetus will develop birth defects due to the use of dental radiographs during pregnancy is considered:

- Very high
- High
- Low
- Very low
- No possibility

9) The possibility that the fetus will develop cancer in the future due to the use of dental radiographs is considered:

- Very high
- High
- Low
- Very low
- No possibility
